# Comparison of brain volumes in episodic and chronic migraine using automated whole-brain volumetry

**DOI:** 10.3389/fneur.2026.1772869

**Published:** 2026-03-16

**Authors:** Halil İbrahim Akçay

**Affiliations:** Department of Neurology, Faculty of Medicine, Niğde Ömer Halisdemir University, Niğde, Türkiye

**Keywords:** brain volumetry, chronic migraine, episodic migraine, volBrain, whole-brain analysis

## Abstract

Migraine chronification is associated with increased clinical burden, yet structural neuroimaging findings remain inconsistent. We compared global and regional brain volumes among patients with episodic migraine (EM), chronic migraine (CM), and healthy controls (HC) using an automated whole-brain volumetric approach. This retrospective study included 58 CM patients, 55 EM patients, and 60 age- and sex-matched HC. Diagnoses were established according to the International Classification of Headache Disorders, 3rd edition (ICHD-3). Structural MRI data acquired on a 1.5-T scanner were processed using the automated volBrain pipeline. Regional brain volumes were compared using one-way ANOVA. To address multiple testing across 220 regions, family-wise error rate (FWER) was controlled with Holm–Bonferroni and false discovery rate (FDR) with Benjamini–Hochberg. Cohen’s *d* values were calculated as exploratory effect sizes. Monthly attack frequency was significantly higher in CM than EM (*p* < 0.001), while disease duration was similar. No significant differences were observed among EM, CM, and HC in global or regional brain volumes after FWER and FDR correction. Regions showing nominal uncorrected differences demonstrated small and inconsistent effect sizes. With rigorous whole-brain correction, automated volumetry did not reveal robust structural differences between episodic and chronic migraine. If present, migraine-related morphometric alterations are likely subtle and methodologically sensitive. Larger longitudinal studies using higher-resolution imaging are needed.

## Introduction

Migraine is a common neurological disorder characterized by recurrent headache attacks that can substantially impair daily functioning. In addition to pain, migraine is frequently accompanied by gastrointestinal, autonomic, cognitive, and emotional symptoms. Current models of migraine pathophysiology involve complex interactions between trigeminovascular activation, altered nociceptive processing, cortical excitability, and neurovascular responses (1).

Episodic migraine (EM) is defined by headache occurring on fewer than 15 days per month, whereas chronic migraine (CM) is characterized by headache on at least 15 days per month for a minimum of 3 months, with migraine features present on a subset of days. The transition from EM to CM (migraine chronification) is heterogeneous and may vary across populations and study designs. Population-based prospective cohort studies have reported chronification rates generally in the range of approximately 2–4% per year, emphasizing that estimates are context-dependent rather than universal (2–4).

Chronic migraine is associated with a higher burden of disability, comorbidity, and reduced quality of life, and it is often more challenging to manage than EM. Rather than reflecting a single linear mechanism, CM is increasingly conceptualized as a multifactorial condition involving central sensitization, altered nociceptive processing, sustained trigeminovascular activity, and dysfunctional corticothalamic modulation. Medication overuse and repeated exposure to acute treatments may further contribute to chronification in susceptible individuals (5). This multifactorial framework also provides a biological rationale for why structural MRI differences between EM and CM may be subtle, variable, or methodologically sensitive.

Neuroimaging studies have investigated structural and functional changes in migraine, implicating brainstem and hypothalamic regions, basal ganglia, and distributed cortical networks. However, reported findings are inconsistent across studies, likely due to heterogeneity in patient phenotyping (e.g., attack burden, comorbidities, medication exposure), imaging protocols, and statistical approaches, including differences in thresholding and multiple-comparison correction. This variability has been highlighted by recent meta-analyses, which suggest that gray matter changes in migraine are heterogeneous and may not constitute a single reliable morphological signature across cohorts (6, 7).

Voxel-based morphometry (VBM) and related morphometric approaches have been widely used to evaluate gray matter volume differences in migraine. While some studies have reported regional gray matter alterations in networks involved in pain processing and multisensory integration, the direction and anatomical localization of these findings differ substantially between studies, and the overall evidence indicates considerable between-study heterogeneity (6–8).

In this study, we investigated global and regional brain volumetric measures in patients with EM and CM as well as healthy controls using an automated whole-brain volumetric approach. Our primary objective was to compare brain volumes across these groups and to explore whether clinical headache characteristics (e.g., frequency and duration) show associations with regional volumetric measures.

## Materials and methods

### Participants

Between December 2019 and November 2023, 58 patients presenting to the neurology outpatient clinic with headache and diagnosed with chronic migraine (CM), 55 patients diagnosed with episodic migraine (EM), and 60 age- and sex-matched healthy controls (HC) were retrospectively included in the study. The control group consisted of individuals presenting with dizziness who had no personal or family history of migraine.

All migraine patients met the International Classification of Headache Disorders, 3rd edition (ICHD-3) criteria (9). The mean number of migraine attacks during the 3 months prior to admission was used for classification. Patients with ≥15 headache days per month in the preceding 3 months were classified as CM, whereas those with <15 headache days per month were classified as EM.

Control subjects were selected from patients presenting with dizziness who had no known history of primary headache disorder, chronic systemic disease, or neurological disease. Individuals with hypercholesterolemia, heart disease, cognitive impairment, other chronic systemic diseases, stroke, substance abuse, hypertension, diabetes mellitus, or other psychiatric or neurological disorders were excluded.

The study protocol was approved by the institutional ethics committee.

### MRI acquisition and automated brain volumetry

Structural MRI data were acquired on a 1.5-T Siemens Aera system using a high-resolution T1-weighted three-dimensional sequence. Imaging parameters included a slice thickness of 1 mm, isotropic voxel size of 1 × 1 × 1 mm, and a field of view of 256 mm. These images were used for automated volumetric analysis with the volBrain pipeline.

T1-weighted 3D images were used for volumetric evaluation. Image data were retrieved from the local Picture Archiving and Communication System (PACS). DICOM (.dcm) files were converted to NIFTI (.nii) format using MRIcron software.

Although conceptually related to morphometric analyses, the present study employed atlas-based automated volumetry using the volBrain pipeline rather than classical voxel-wise VBM. Automated volumetric approaches have demonstrated good agreement with established neuroimaging pipelines and provide robust regional volume estimation in clinical datasets (10).

### volBrain analysis

Anonymized and compressed T1-weighted MRI images in NIFTI format were uploaded to the volBrain online system for fully automated whole-brain volumetric analysis. The volBrain pipeline performs multiple preprocessing steps, including denoising, inhomogeneity correction, affine registration, intensity normalization, and automated segmentation of brain tissues and substructures.

Following processing, the system generated detailed volumetric reports including gray matter, white matter, cerebrospinal fluid, and regional brain volumes, all expressed in cubic centimeters (11).

All volumetric measurements were obtained using the fully automated volBrain pipeline. Given the automated nature of the volBrain pipeline, additional manual visual quality control was not systematically performed; however, all images successfully passed the internal quality checks of the volBrain system.

### Statistical analysis

The required sample size was calculated as 171 subjects using G*Power 3.1.9.6 with 95% power and a 0.05 margin of error, based on parameters reported by Chen et al. (12). The final cohort (*n* = 173) met this requirement.

Statistical analyses were performed using IBM SPSS version 26.0 (IBM Corp., Armonk, NY, United States). Normality of quantitative variables was assessed using the Kolmogorov–Smirnov test, histograms, and Q–Q plots. Homogeneity of variances was evaluated using Levene’s test.

Categorical variables were analyzed using the chi-square test or Fisher’s exact test as appropriate. Because monthly attack frequency, attack duration, and disease duration were not normally distributed, comparisons between migraine groups were performed using the Mann–Whitney *U* test. Age comparisons across all three groups were performed using the Kruskal–Wallis test.

Regional brain volumes were compared among EM, CM, and HC groups using one-way ANOVA. In addition to *p*-values, effect sizes (Cohen’s *d*) were calculated for key comparisons and reported as exploratory measures to facilitate comparison with prior literature.

Given the large number of regional comparisons (*n* = 220), multiple-comparison correction was performed using R version 4.3.2 (13). Family-wise error rate (FWER) was controlled using the Holm–Bonferroni method (14), and false discovery rate (FDR) was controlled using the Benjamini–Hochberg procedure (15) via the p.adjust function. This dual-correction strategy was applied to minimize Type I error while preserving interpretability of exploratory findings.

**Figure 1:**
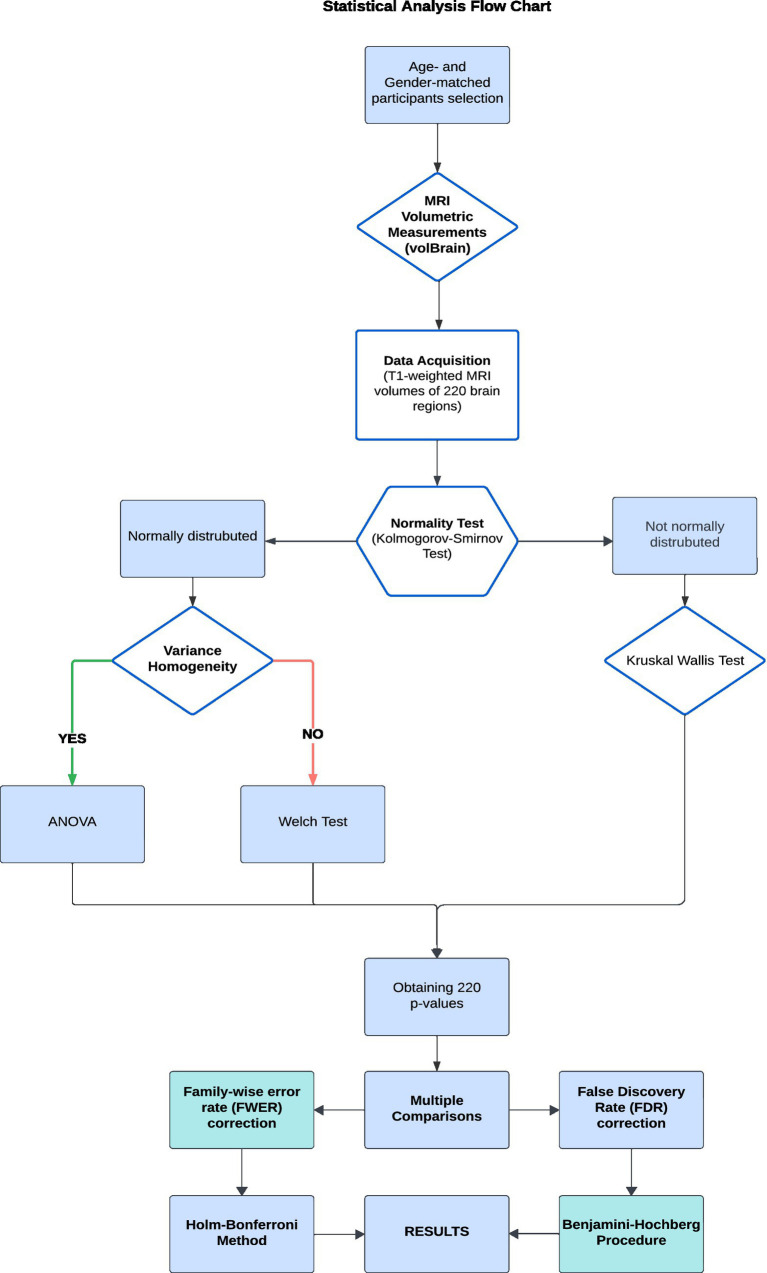
Statistical analysis of data.

All statistical analysis steps are summarized in the flowchart shown in Figure 1.

## Results

Headache groups were compared in terms of monthly attack frequency, disease duration, and attack duration. The median monthly attack frequency was 4 in the episodic migraine (EM) group and 30 in the chronic migraine (CM) group, demonstrating a statistically significant difference between the groups (*p* < 0.001). With respect to attack duration, the median value was 2 in the EM group and 1 in the CM group, indicating significantly longer attacks in the EM group (*p* < 0.001). Disease duration was comparable between the two migraine subgroups, and no statistically significant difference was detected (*p* = 0.935). A detailed summary of these comparisons is provided in Table 1.

**Table 1:** Comparison of migraine groups in terms of monthly attack frequency, disease duration, and attack duration.

Variable	EM	CM	Test St.	p*
	Median (min–max)	Median (min–max)		
Number of attacks per month	4 (1–12)	30 (8–30)	11.5	**<0.001**
Duration of headache	7 (2–32)	7 (2–37)	1,581	0.935
Attack duration	2 (1–3)	1 (1–2)	742.5	**<0.001**

*Mann–Whitney *U* test.

EM, episodic migraine; CM, chronic migraine. Bold values indicate statistically significant differences (*p* < 0.05).

There was no statistically significant difference in patient age among the healthy control (HC), EM, and CM groups (*p* = 0.383) (Table 2). Similarly, comparison of clinical characteristics between EM and CM groups revealed no significant differences in headache-related features (Table 3), indicating that the migraine subgroups were broadly comparable apart from expected differences in attack frequency and duration.

**Table 2:** Comparison of age among healthy controls, episodic migraine, and chronic migraine groups.

Group	HC	EM	CM	Test St.	p*
	Median (min–max)	Median (min–max)	Median (min–max)		
Age of patient	33 (18–58)	31 (18–60)	30 (18–65)	1.919	0.383

*Kruskal–Wallis test.

HC, healthy control; EM, episodic migraine; CM, chronic migraine.

**Table 3:** Comparison of clinical characteristics between episodic and chronic migraine groups.

Variable	Group		Total (*n*, %)	*p*
	EM (*n*, %)	CM (*n*, %)		
Increase with physical activity				
No	4 (44.4)	5 (55.6)	9 (100)	1.0**
Yes	51 (92.7)	53 (91.4)	104 (92)	
Gender				
Female	54 (98.2)	56 (96.6)	110 (97.3)	1.0**
Male	1 (1.8)	2 (3.4)	3 (2.7)	
Vomiting				
No	35 (63.6)	44 (75.9)	79 (69.9)	0.157*
Yes	20 (36.4)	14 (24.1)	34 (30.1)	
Phonophobia				
No	3 (5.5)	5 (8.6)	8 (7.1)	0.717**
Yes	52 (94.5)	53 (91.4)	105 (92.9)	
Photophobia				
No	5 (9.1)	9 (15.5)	14 (12.4)	0.453***
Yes	50 (90.9)	49 (84.5)	99 (87.6)	
Osmophobia				
No	17 (30.9)	18 (31)	35 (31)	0.989*
Yes	55 (100)	58 (100)	113 (100)	
Vertigo				
No	37 (67.3)	36 (62.1)	73 (64.6)	0.563*
Yes	18 (32.7)	22 (37.9)	40 (35.4)	
Menstrual exacerbation				
No	19 (34.5)	26 (44.8)	45 (39.8)	0.261*
Yes	32 (58.2)	25 (43.1)	57 (50.4)	
Not applicable	4 (7.3)	7 (12.1)	11 (9.7)	
Family history				
No	27 (49.1)	28 (48.3)	55 (48.7)	0.931*
Yes	28 (50.9)	30 (51.7)	58 (51.3)	

*Pearson Chi Square Test, **Fisher Exact Test, ***Continuity Correction.

EM, episodic migraine; CM, chronic migraine.

In the global volumetric assessment, no statistically significant differences were observed among HC, EM, and CM groups in total white matter, cortical gray matter, subcortical gray matter, cerebellar gray matter, cerebellar vermis, or brainstem volumes. Hemispheric analyses of these global brain measures likewise did not reveal any significant intergroup differences. These findings are summarized in Supplementary Table S1.

Evaluation of basal ganglia substructures similarly demonstrated no significant volumetric differences among the three groups. Volumetric comparisons of the caudate nucleus, putamen, globus pallidus, and related substructures were statistically comparable across HC, EM, and CM groups. Detailed results of these analyses are presented in Supplementary Tables S1, S2.

At the lobar and gyral levels, several regions demonstrated apparent group differences in the initial uncorrected analyses, including the frontal pole (total, right, and left), right orbital inferior frontal gyrus, planum temporale (total and left), and left parietal operculum (Table 4). To aid interpretation, effect size estimates (Cohen’s *d*) for key comparisons are provided in Supplementary Table S3 as exploratory measures.

**Table 4:** Comparison of brain volumes.

Volumes (cm^3^)	HC		EM		CM		*p*
	Mean ± SD	Median (min-max)	Mean ± SD	Median (min-max)	Mean ± SD	Median (min-max)	
Ventral DC total	8.6 ± 1.0	8.6 (6.2–10.4)	8.7 ± 1	8.7 (6.4–11.7)	8.5 ± 0.9	8.6 (6.6–10.7)	0.644*
Ventral DC right	4.3 ± 0.5	4.3 (3.0–5.3)	4.3 ± 0.5	4.3 (3.1–6.2)	4.2 ± 0.5	4.2 (3.1–5.4)	0.615*
Ventral DC left	4.3 ± 0.5	4.2 (3.2–5.3)	4.4 ± 0.5	4.4 (3.3–5.9)	4.3 ± 0.5	4.3 (3.3–5.4)	0.608*
Frontal total	156.1 ± 25.3	154 (92.6–219.5)	164.1 ± 26.6	162.2 (95.6–253.6)	158.4 ± 20.9	155.6 (124.0–226.0)	0.232**
Frontal right	78.7 ± 12.8	77.7 (47.1–111.3)	82.6 ± 13.5	82.4 (47.1–128)	79.8 ± 10.5	78.3 (61.1–115.1)	0.231*
Frontal left	77.4 ± 12.6	76.4 (45.6–108.1)	81.5 ± 13.3	79.7 (48.5–125.6)	78.6 ± 10.6	76.7 (61.2–110.9)	0.248**
Frontal pole total	5.9 ± 1.4^a^	6.1 (1.6–8.2)	6.7 ± 1.5^bc^	6.6 (3–9.3)	6.3 ± 1.3^ac^	6.2 (3.8–9.2)	0.013*
Frontal pole right	3.1 ± 0.8^a^	3.1 (0.8–4.4)	3.4 ± 0.8^bc^	3.4 (1.3–5.3)	3.2 ± 0.7^ac^	3.2 (1.7–5.3)	0.041*
Frontal pole left	2.9 ± 0.7^a^	3.0 (0.8–4.1)	3.3 ± 0.7^bc^	3.2 (1.7–4.7)	3.1 ± 0.7^ac^	3.0 (2.0–4.5)	0.008*
Gyrus rectus total	3.4 ± 0.7	3.4 (1.2–4.9)	3.7 ± 0.9	3.6 (1.7–6.0)	3.5 ± 0.8	3.4 (2.0–6.1)	0.133*
Gyrus rectus right	1.8 ± 0.4	1.8 (0.8–2.6)	1.9 ± 0.5	1.8 (0.9–2.9)	1.8 ± 0.4	1.8 (1.1–3.2)	0.160*
Gyrus rectus left	1.6 ± 0.4	1.6 (0.4–2.4)	1.8 ± 0.5	1.7 (0.8–3.2)	1.7 ± 0.4	1.6 (0.8–2.9)	0.261**
Opercular inf. Frontal gyrus total	5.5 ± 1.2	5.3 (3.2–8.3)	5.9 ± 1.2	5.7 (4.1–11.4)	5.7 ± 1.0	5.5 (3.9–8.2)	0.288**
Opercular inf. Frontal gyrus right	2.9 ± 0.7	2.7 (1.8–5.0)	3.0 ± 0.6	3.0 (2.0–5.3)	2.9 ± 0.6	2.8 (1.7–4.2)	0.274**
Opercular inf. Frontal gyrus left	2.6 ± 0.6	2.6 (1.4–3.9)	2.9 ± 0.7	2.8 (1.9–6.1)	2.8 ± 0.5	2.8 (1.8–4.3)	0.251**
Orbital inf. Frontal gyrus total	1.9 ± 0.7	1.9 (0.9–3.6)	2.1 ± 0.5	2.1 (0.9–3.2)	1.9 ± 0.5	1.8 (1.0–3.2)	0.055**
Orbital inf. Frontal gyrus right	0.9 ± 0.4	0.8 (0.3–2.0)^ab^	1.0 ± 0.3	1.0 (0.5–1.7)^a^	0.9 ± 0.4	0.8 (0.3–2.1)^b^	0.031**
Orbital inf. Frontal gyrus left	1.0 ± 0.4	0.9 (0.3–2.0)	1.1 ± 0.4	1.1 (0.4–2.2)	1.0 ± 0.4	1.0 (0.4–1.8)	0.376*
Triangular inf. Frontal gyrus total	5.7 ± 1.1	5.7 (3.9–9.3)	6.1 ± 1.4	5.9 (3.8–11.2)	5.8 ± 1.1	5.8 (3.4–8.3)	0.180*
Triangular inf. Frontal gyrus right	2.9 ± 0.7	2.8 (1.7–4.9)	3.1 ± 0.8	3.0 (1.7–5.5)	3.0 ± 0.6	2.9 (1.7–4.5)	0.295**
Triangular inf. Frontal gyrus left	2.8 ± 0.6	2.7 (1.8–4.4)	3.0 ± 0.7	3.0 (1.9–5.8)	2.8 ± 0.6	2.8 (1.4–3.8)	0.346**
Medial frontal cortex total	2.5 ± 0.6	2.5 (0.9–3.9)	2.7 ± 0.7	2.8 (0.8–4.9)	2.5 ± 0.5	2.5 (1.5–3.7)	0.107*
Medial frontal cortex right	1.3 ± 0.3	1.3 (0.4–1.9)	1.4 ± 0.3	1.4 (0.4–2.2)	1.3 ± 0.3	1.3 (0.8–1.8)	0.215*
Medial frontal cortex left	1.2 ± 0.3	1.2 (0.4–2.1)	1.3 ± 0.4	1.4 (0.4–2.7)	1.2 ± 0.3	1.2 (0.6–1.9)	0.060*
Middle frontal gyrus total	35.1 ± 5.8	35.2 (21.6–49.6)	36.7 ± 6.7	36.0 (20.0–62.6)	35.5 ± 5.3	34.4 (26–52.1)	0.316*
Middle frontal gyrus right	17.9 ± 3.0	17.8 (10.3–26.4)	18.8 ± 3.5	18.4 (9.4–31.5)	18.1 ± 2.7	17.9 (13.3–27.4)	0.258*
Middle frontal gyrus left	17.2 ± 2.9	17.5 (11.3–23.3)	17.9 ± 3.3	17.9 (10.6–31.1)	17.5 ± 2.7	17.2 (12.7–24.7)	0.411*
Anterior orbital gyrus total	3.5 ± 0.8	3.6 (1.1–5.6)	3.7 ± 0.9	3.7 (2.2–7.1)	3.7 ± 0.7	3.6 (2.4–6.1)	0.353*
Anterior orbital gyrus right	1.8 ± 0.5	1.8 (0.5–3.0)	2.0 ± 0.5	1.9 (1.1–3.7)	1.9 ± 0.4	1.9 (1.1–3.1)	0.119*
Anterior orbital gyrus left	1.7 ± 0.4	1.8 (0.5–2.9)	1.8 ± 0.5	1.7 (0.9–3.4)	1.8 ± 0.4	1.8 (1.2–3.1)	0.826*
Lateral orbital gyrus total	3.1 ± 0.8	3.2 (1.2–5.0)	3.4 ± 0.8	3.2 (2.0–5.5)	3.2 ± 0.8	3.2 (1.8–4.9)	0.085*
Lateral orbital gyrus right	1.6 ± 0.4	1.5 (0.6–2.7)	1.7 ± 0.4	1.7 (0.9–2.9)	1.6 ± 0.4	1.6 (0.8–2.7)	0.199*
Lateral orbital gyrus left	1.5 ± 0.4	1.4 (0.6–2.3)	1.7 ± 0.4	1.6 (0.9–2.8)	1.6 ± 0.4	1.5 (0.7–2.7)	0.089*
Medial orbital gyrus total	7.4 ± 1.6	7.2 (2.9–11.5)	7.7 ± 1.5	7.4 (4.7–11.9)	7.5 ± 1.4	7.5 (4.6–11.9)	0.591*
Medial orbital gyrus right	3.7 ± 0.9	3.5 (1.5–5.8)	3.8 ± 0.8	3.7 (2.4–6.1)	3.7 ± 0.8	3.7 (2.2–6.1)	0.709*
Medial orbital gyrus left	3.7 ± 0.8	3.7 (1.4–5.8)	3.9 ± 0.7	3.8 (2.3–5.8)	3.7 ± 0.7	3.7 (2.4–5.7)	0.485*
Posterior orbital gyrus total	5.6 ± 1.1	5.5 (2.8–8.4)	5.8 ± 1.2	5.6 (3.6–9.3)	5.6 ± 0.7	5.6 (4.2–7.5)	0.901**
Posterior orbital gyrus right	2.7 ± 0.6	2.7 (1.1–4.1)	2.8 ± 0.6	2.7 (1.8–5.1)	2.8 ± 0.4	2.8 (2.0–3.7)	0.855**
Posterior orbital gyrus left	2.8 ± 0.5	2.8 (1.7–4.2)	2.9 ± 0.6	2.9 (1.7–4.7)	2.8 ± 0.4	2.8 (2.0–3.9)	0.756**
Precentral gyrus total	22.5 ± 3.5	21.4 (17.2–33.7)	22.8 ± 3.8	22.4 (13.5–32.4)	22.6 ± 3.2	21.8 (17.2–31.1)	0.685**
Precentral gyrus right	11.2 ± 1.7	10.6 (8.3–17.3)	11.3 ± 1.9	11.0 (6.9–16.4)	11.3 ± 1.7	11.1 (8.2–16.2)	0.837**
Precentral gyrus left	11.3 ± 1.9	10.7 (8.3–16.9)	11.5 ± 2.0	11.3 (6.6–17.5)	11.3 ± 1.6	11.1 (8.4–15.6)	0.728**
Precentral gyrus medial segment total	5.1 ± 0.9	5.0 (3.2–8.0)	5.3 ± 0.9	5.3 (2.4–7.7)	5.1 ± 0.8	4.9 (3.6–7.4)	0.135**
Precentral gyrus medial segment right	2.6 ± 0.5	2.5 (1.6–3.7)	2.6 ± 0.5	2.7 (1.1–4.0)	2.5 ± 0.5	2.4 (1.5–3.7)	0.129**
Precentral gyrus medial segment left	2.6 ± 0.5	2.6 (1.6–4.6)	2.7 ± 0.5	2.7 (1.3–3.8)	2.6 ± 0.4	2.5 (1.7–3.8)	0.398*
Subcallosal area total	2.5 ± 0.7	2.5 (1.2–4.0)	2.6 ± 0.6	2.5 (1.6–4.2)	2.5 ± 0.6	2.5 (1.5–4.5)	0.886**
Subcallosal area right	1.3 ± 0.4	1.3 (0.5–2.2)	1.3 ± 0.3	1.3 (0.8–2.2)	1.3 ± 0.3	1.3 (0.8–2.3)	0.894**
Subcallosal area left	1.3 ± 0.3	1.2 (0.7–2.0)	1.3 ± 0.3	1.2 (0.8–2.1)	1.2 ± 0.3	1.2 (0.7–2.2)	0.783**
Sup. frontal gyrus total	25.8 ± 4.7	25.3 (13.3–38.4)	27.3 ± 4.4	27.0 (17.6–36.3)	26.3 ± 4.4	25.4 (19.9–40.6)	0.189**
Sup. frontal gyrus right	12.8 ± 2.3	12.5 (7.0–18.2)	13.4 ± 2.2	13.2 (8.7–18)	13.0 ± 2.1	12.6 (9.5–18.9)	0.210**
Sup. frontal gyrus left	13.0 ± 2.6	12.8 (6.4–20.2)	13.8 ± 2.4	13.7 (8.9–18.5)	13.3 ± 2.4	12.9 (9.5–21.7)	0.217**
Sup. frontal gyrus medial segment total	11.3 ± 1.8	11.4 (5.8–14.8)	12.0 ± 2.1	12.1 (6.3–18.1)	11.5 ± 1.7	11.4 (8.4–15.4)	0.082*
Sup. frontal gyrus medial segment right	5.9 ± 1.0	5.8 (3.2–7.8)	6.2 ± 1.2	6.3 (3.2–9.6)	6.0 ± 0.9	5.9 (4.2–8.0)	0.179*
Sup. frontal gyrus medial segment left	5.4 ± 1.0	5.4 (2.6–7.5)	5.8 ± 1.1	5.7 (3.2–8.5)	5.5 ± 0.9	5.4 (3.9–7.9)	0.062*
Supplementary motor cortex total	9.2 ± 1.5	9.3 (5.8–12.0)	9.5 ± 1.5	9.3 (5.5–13.2)	9.3 ± 1.3	9.3 (6.8–12.7)	0.638*
Supplementary motor cortex right	4.6 ± 0.8	4.4 (2.9–6.0)	4.7 ± 0.9	4.6 (2.5–6.6)	4.6 ± 0.7	4.6 (3.6–6.3)	0.742*
Supplementary motor cortex left	4.7 ± 0.8	4.6 (2.8–6.2)	4.8 ± 0.8	4.7 (3.0–7.2)	4.7 ± 0.7	4.6 (3.1–6.6)	0.576*
Temporal total	98.7 ± 16.7	95.9 (68.9–141.7)	102.3 ± 17.3	98.9 (75.1–160.4)	97.3 ± 12.8	96.3 (72.0–128.1)	0.362**
Temporal right	49.4 ± 8.9	47.6 (35.4–72.0)	50.9 ± 9.1	48.7 (37.0–81.1)	48.4 ± 6.5	47.9 (34.5–63.0)	0.269*
Temporal left	49.3 ± 8.0	48.0 (33.6–69.7)	51.4 ± 8.7	49.2 (38.1–79.3)	48.9 ± 6.4	48.4 (33.9–65.6)	0.384**
Fusiform gyrus total	14.1 ± 2.8	13.9 (9.1–20.7)	14.7 ± 2.8	14.2 (9.1–23.2)	14.1 ± 2.2	13.9 (9.9–19.6)	0.395*
Fusiform gyrus right	6.9 ± 1.4	6.6 (4.6–10.3)	7.1 ± 1.5	7.0 (4.4–10.8)	6.8 ± 1.1	6.8 (4.5–9.5)	0.565**
Fusiform gyrus left	7.2 ± 1.4	7.2 (4.5–10.6)	7.6 ± 1.4	7.1 (4.7–12.4)	7.3 ± 1.2	7.1 (4.7–10.8)	0.453**
Planum polare total	3.3 ± 0.6	3.3 (2.3–4.9)	3.5 ± 0.5	3.5 (2.3–4.8)	3.4 ± 0.4	3.3 (2.5–4.4)	0.137**
Planum polare right	1.6 ± 0.3	1.5 (1.0–2.6)	1.7 ± 0.3	1.6 (1.1–2.4)	1.6 ± 0.2	1.6 (1.1–2.2)	0.067**
Planum polare left	1.7 ± 0.3	1.7 (1.1–2.7)	1.8 ± 0.3	1.8 (1.2–2.6)	1.7 ± 0.2	1.7 (1.3–2.4)	0.540*
Planum temporale total	3.4 ± 0.7^cd^	3.3 (1.6–5.3)	3.5 ± 0.7^ac^	3.5 (1.6–5.2)	3.2 ± 0.7^bd^	3.3 (1.7–4.7)	0.044*
Planum temporale right	1.5 ± 0.4	1.5 (0.7–2.8)	1.5 ± 0.4	1.5 (0.3–2.6)	1.5 ± 0.3	1.4 (0.7–2.2)	0.562**
Planum temporale left	1.9 ± 0.4	1.8 (0.9–3.0)^ab^	2.0 ± 0.4	2.0 (1.0–2.8)^a^	1.8 ± 0.4	1.8 (0.8–2.6)^b^	0.032**
Inf. temporal gyrus total	19.6 ± 3.8	18.8 (9.8–29.0)	20.2 ± 3.9	20 (13.5–31.0)	19.1 ± 2.8	19.2 (13.6–25.4)	0.257*
Inf. temporal gyrus right	9.6 ± 2.0	9.2 (5.4–14.3)	9.8 ± 2.0	9.7 (5.9–15.9)	9.3 ± 1.5	9.2 (6.4–13.1)	0.344*
Inf. temporal gyrus left	9.9 ± 2.0	9.7 (4.4–14.7)	10.4 ± 2.0	10.1 (7.3–15.1)	9.8 ± 1.5	10.0 (7.0–13.2)	0.410**
Middle temporal gyrus total	25.1 ± 4.9	24.3 (15.8–38.4)	25.8 ± 5.2	25.8 (15.9–45.6)	24.8 ± 4.1	24.1 (17.4–35.4)	0.464*
Middle temporal gyrus right	12.8 ± 2.7	12.2 (7.9–20.5)	13.2 ± 3.0	13.1 (7.2–24.6)	12.5 ± 2.1	12.4 (8.4–17.6)	0.600**
Middle temporal gyrus left	12.2 ± 2.3	12.1 (7.7–17.8)	12.7 ± 2.6	12.3 (7.6–21.0)	12.2 ± 2.2	12.2 (7.2–17.8)	0.511*
Sup. temporal gyrus total	13.4 ± 2.3	12.9 (8.8–18.4)	13.9 ± 2.3	13.6 (9.9–21.9)	13.4 ± 1.8	13.2 (9.3–16.9)	0.596**
Sup. temporal gyrus right	6.8 ± 1.3	6.6 (4.4–9.9)	7.0 ± 1.4	6.8 (4.6–11.1)	6.7 ± 1.0	6.6 (4.1–8.5)	0.605**
Sup. temporal gyrus left	6.6 ± 1.0	6.4 (4.2–8.9)	6.8 ± 1.1	6.8 (5.0–10.7)	6.7 ± 1.0	6.6 (4.3–9.0)	0.432*
Transverse temporal gyrus total	2.9 ± 0.6	2.9 (1.8–4.5)	2.8 ± 0.5	2.9 (1.6–4.0)	2.8 ± 0.6	2.7 (1.9–4.1)	0.547*
Transverse temporal gyrus right	1.4 ± 0.3	1.3 (0.8–2.1)	1.3 ± 0.3	1.3 (0.8–2.0)	1.4 ± 0.3	1.3 (0.8–2.1)	0.972*
Transverse temporal gyrus left	1.6 ± 0.4	1.6 (0.9–2.6)	1.5 ± 0.3	1.5 (0.6–2.1)	1.4 ± 0.3	1.3 (0.9–2.2)	0.158**
Temporal pole total	16.9 ± 3.4	16.3 (8.5–24.9)	17.9 ± 3.8	16.9 (11.7–26.6)	16.6 ± 2.8	16.2 (11.7–24.0)	0.244**
Temporal pole right	8.7 ± 1.8	8.6 (4.6–13.2)	9.3 ± 2.0	9.0 (5.9–13.5)	8.6 ± 1.5	8.5 (5.8–12.3)	0.106*
Temporal pole left	8.2 ± 1.7	8 (3.9–12.0)	8.6 ± 1.9	8.1 (5.1–13.2)	8.0 ± 1.5	7.9 (3.4–12.8)	0.294**
Parietal total	94.8 ± 14.0	93.1 (66.7–131.9)	98.5 ± 16.7	97.5 (51.7–154.3)	96.0 ± 12.7	94.4 (75.2–129.2)	0.341**
Parietal right	47.3 ± 7.0	45.9 (34.7–65.4)	49.0 ± 8.6	49.2 (23–78.7)	47.8 ± 6.5	46.9 (36.0–66.1)	0.388**
Parietal left	47.5 ± 7.1	46.9 (32.0–68.0)	49.5 ± 8.3	48.3 (28.7–75.6)	48.3 ± 6.5	47.8 (37.6–63.0)	0.402**
Angular gyrus total	19.5 ± 3.2	19.2 (13.7–27.0)	20.4 ± 4.2	19.8 (9.9–37.2)	19.7 ± 3.4	19.5 (12.7–27.7)	0.342**
Angular gyrus right	10.4 ± 1.8	10.2 (7.3–15.1)	10.6 ± 2.4	10.5 (4.1–19.8)	10.3 ± 1.8	10.1 (6.5–15.3)	0.680**
Angular gyrus left	9.1 ± 1.6	9.0 (6.0–13.1)	9.8 ± 2.0	9.6 (5.9–17.4)	9.3 ± 1.8	8.9 (6.0–14.9)	0.148*
Postcentral gyrus total	17.8 ± 2.5	17.4 (13.0–24.6)	18.3 ± 3.1	18.3 (11.3–28.1)	17.7 ± 2.6	17.6 (13.0–25.2)	0.483*
Postcentral gyrus right	8.7 ± 1.4	8.4 (6.5–12.8)	8.9 ± 1.6	8.8 (5.0–14.3)	8.7 ± 1.3	8.5 (6.0–12.5)	0.607*
Postcentral gyrus left	9.1 ± 1.3	9.0 (6.2–11.9)	9.4 ± 1.7	9.2 (6.3–13.8)	9.1 ± 1.4	9.1 (6.1–12.7)	0.416*
Postcentral gyrus medial segment total	1.7 ± 0.4	1.7 (0.8–2.5)	1.7 ± 0.4	1.8 (0.1–2.5)	1.7 ± 0.3	1.7 (0.5–2.6)	0.749*
Postcentral gyrus medial segment right	0.9 ± 0.2	0.9 (0.3–1.4)	0.8 ± 0.2	0.9 (0.0–1.4)	0.9 ± 0.2	0.9 (0.2–1.4)	0.968*
Postcentral gyrus medial segment left	0.8 ± 0.2	0.8 (0.4–1.6)	0.9 ± 0.3	0.9 (0.0–1.7)	0.9 ± 0.2	0.9 (0.3–1.5)	0.327*
Precuneus total	21.4 ± 3.9	20.9 (13.6–31.9)	22.4 ± 3.9	21.6 (12.6–32.0)	21.4 ± 2.9	21.5 (15.8–27.5)	0.326**
Precuneus right	10.7 ± 2.0	10.5 (7.1–16.2)	11.3 ± 2.0	11.0 (5.9–16.8)	10.8 ± 1.5	10.8 (7.4–14.7)	0.221**
Precuneus left	10.7 ± 2.0	10.3 (6.5–16.0)	11.1 ± 2.0	10.6 (6.7–15.2)	10.7 ± 1.5	10.6 (8.0–13.8)	0.479**
Sup. parietal lobule total	19.6 ± 2.8	19.0 (12.6–27.5)	20.2 ± 3.9	20.2 (4.2–29.7)	20.2 ± 2.9	19.5 (15.1–27.2)	0.411**
Sup. parietal lobule right	9.5 ± 1.4	9.3 (6.6–13.0)	9.9 ± 2.1	9.6 (1.3–14.7)	9.7 ± 1.5	9.4 (7.4–13.5)	0.589*
Sup. parietal lobule left	10.1 ± 1.5	9.9 (6.0–14.5)	10.4 ± 1.9	10.2 (2.9–15.8)	10.5 ± 1.6	10.3 (7.4–14.3)	0.302**
Supramarginal gyrus total	14.9 ± 2.7	14.3 (11.1–22.1)	15.6 ± 2.9	15.5 (9.9–24.8)	15.2 ± 2.4	15.0 (10.3–21.1)	0.348**
Supramarginal gyrus right	7.2 ± 1.3	6.9 (5.0–10.8)	7.6 ± 1.5	7.3 (5.0–12.5)	7.4 ± 1.3	7.2 (4.5–11.5)	0.264**
Supramarginal gyrus left	7.7 ± 1.5	7.4 (5.2–11.5)	8.0 ± 1.6	7.7 (4.7–12.3)	7.8 ± 1.2	7.8 (5.5–10.6)	0.637**
Occipital total	60.5 ± 9.8	59.7 (42.8–84.3)	61.6 ± 10.8	60.5 (45.5–97.5)	60.4 ± 8.7	59.8 (40–77.7)	0.765*
Occipital right	29.5 ± 5.0	29.3 (18.9–40.9)	30.0 ± 5.7	29.4 (18.5–48.2)	29.2 ± 4.3	28.8 (20.2–38.5)	0.815**
Occipital left	31.0 ± 5.0	30.3 (20.9–43.4)	31.6 ± 5.5	31.0 (22.3–49.3)	31.2 ± 4.9	30.8 (19.8–40.8)	0.833*
Calcarine cortex total	4.5 ± 1.3	4.2 (2.1–7.5)	4.6 ± 1.5	4.4 (2.2–11.0)	4.5 ± 1.3	4.3 (2.5–9.3)	0.992**
Calcarine cortex left	2.3 ± 0.7	2.2 (0.9–4.0)	2.3 ± 0.8	2.2 (1.0–5.1)	2.3 ± 0.8	2.2 (0.9–4.7)	0.993*
Cuneus total	6.7 ± 1.1	6.6 (4.8–10.1)	6.9 ± 1.0	6.9 (4.0–8.8)	6.7 ± 1.0	6.6 (4.5–9.9)	0.623*
Cuneus right	3.4 ± 0.7	3.3 (2.3–5.2)	3.4 ± 0.6	3.5 (2.0–4.6)	3.3 ± 0.6	3.3 (2.2–5.0)	0.543*
Cuneus left	3.4 ± 0.6	3.3 (2.3–4.9)	3.4 ± 0.6	3.5 (2.0–4.5)	3.4 ± 0.6	3.4 (2.2–4.9)	0.770*
Lingual gyrus total	15.2 ± 2.2	14.8 (10.3–19.9)	15.4 ± 2.2	15.3 (11.3–22.3)	15.2 ± 2.2	14.8 (11.0–21.4)	0.869**
Lingual gyrus right	7.5 ± 1.2	7.4 (4.9–9.8)	7.6 ± 1.0	7.5 (5.7–10.4)	7.4 ± 1.1	7.3 (5.4–11.4)	0.635**
Lingual gyrus left	7.7 ± 1.2	7.4 (5.2–11.1)	7.8 ± 1.3	7.6 (5.4–11.8)	7.8 ± 1.3	7.6 (5.2–10.6)	0.872**
Occipital fusiform gyrus total	4.9 ± 0.9	4.8 (3.2–7.0)	5.0 ± 1.0	4.9 (3.1–8.5)	5.0 ± 0.9	4.9 (3.2–7.5)	0.780**
Occipital fusiform gyrus right	2.3 ± 0.5	2.3 (1.4–3.3)	2.5 ± 0.6	2.4 (1.3–4.0)	2.4 ± 0.5	2.3 (1.4–3.7)	0.522**
Occipital fusiform gyrus left	2.5 ± 0.6	2.6 (1.1–4.1)	2.6 ± 0.6	2.4 (1.6–4.6)	2.6 ± 0.5	2.6 (1.5–3.9)	0.646*
Inf. occipital gyrus total	10.0 ± 2.1	9.9 (5.5–16.9)	10.0 ± 2.2	10.0 (6.6–18.0)	9.8 ± 1.7	9.9 (5.5–12.9)	0.906**
Inf. occipital gyrus right	4.8 ± 1.1	4.7 (2.2–8.1)	4.9 ± 1.3	4.7 (2.0–8.5)	4.7 ± 0.9	4.7 (2.8–6.8)	0.994**
Inf. occipital gyrus left	5.2 ± 1.1	5.1 (3.3–8.8)	5.1 ± 1.1	5.1 (3.4–9.5)	5.0 ± 1.0	5.0 (2.6–7.4)	0.680*
Middle occipital gyrus total	8.9 ± 1.8	8.7 (5.4–14.7)	9.4 ± 2.5	9.2 (4.8–19.5)	9.1 ± 1.8	8.7 (5.7–13.7)	0.431**
Middle occipital gyrus right	4.2 ± 0.9	4.0 (2.3–7.1)	4.4 ± 1.4	4.2 (1.5–9.5)	4.2 ± 0.9	4.2 (2.7–6.3)	0.660**
Middle occipital gyrus left	4.7 ± 1.0	4.6 (3.1–7.6)	5.0 ± 1.3	4.8 (3.1–10.0)	4.9 ± 1.1	4.8 (3.0–7.5)	0.307**
Sup. occipital gyrus total	6.7 ± 1.1	6.7 (4.2–9.8)	6.8 ± 1.3	6.8 (4.2–10.7)	6.7 ± 0.9	6.7 (4.4–9.0)	0.704***
Sup. occipital gyrus right	3.6 ± 0.7	3.5 (1.9–5.5)	3.6 ± 0.9	3.6 (2.0–6.4)	3.4 ± 0.5	3.4 (2.2–4.5)	0.434***
Sup. occipital gyrus left	3.2 ± 0.5	3.2 (2.0–4.4)	3.3 ± 0.6	3.2 (1.9–4.6)	3.2 ± 0.6	3.2 (2.0–4.9)	0.678*
Occipital pole total	3.6 ± 1.0	3.7 (1.1–5.9)	3.5 ± 1.0	3.5 (1.3–6.1)	3.5 ± 1.1	3.5 (0.6–6.4)	0.789*
Occipital pole right	1.6 ± 0.5	1.6 (0.1–3.0)	1.5 ± 0.5	1.4 (0.7–2.9)	1.5 ± 0.5	1.4 (0.2–3.2)	0.459*
Occipital pole left	2.0 ± 0.6	2.1 (0.8–3.6)	2.0 ± 0.6	2.0 (0.6–3.4)	2.0 ± 0.6	1.9 (0.4–3.7)	0.996*
Limbic total	41.8 ± 7.5	41.1 (22.3–61.1)	43.3 ± 7.1	42.7 (30.9–64.7)	42.0 ± 5.1	41.8 (33.2–56.2)	0.425*
Limbic right	20.5 ± 3.7	20.3 (10.4–29.8)	21.2 ± 3.5	20.9 (15.3–31.6)	20.6 ± 2.6	20.5 (15.7–27.8)	0.527*
Limbic left	21.2 ± 3.9	20.8 (11.9–31.3)	22.1 ± 3.7	21.4 (15.6–33.1)	21.3 ± 2.7	21.4 (16.7–28.3)	0.353*
Entorhinal area total	3.5 ± 0.8	3.4 (1.5–5.8)	3.6 ± 0.6	3.5 (2.5–6.1)	3.5 ± 0.6	3.5 (2.1–5.2)	0.696**
Entorhinal area right	1.8 ± 0.4	1.8 (0.8–3.1)	1.8 ± 0.3	1.8 (1.3–2.5)	1.8 ± 0.3	1.8 (1.2–2.5)	0.800**
Entorhinal area left	1.7 ± 0.4	1.7 (0.8–2.8)	1.8 ± 0.4	1.7 (1.2–3.7)	1.7 ± 0.3	1.7 (0.6–2.7)	0.678**
Anterior cingulate gyrus total	12.6 ± 2.5	12.3 (4.9–18.7)	13.1 ± 2.2	13.1 (7.8–19.9)	12.6 ± 1.7	12.5 (9.7–16.4)	0.342*
Anterior cingulate gyrus right	6.0 ± 1.3	6.0 (2.2–9.4)	6.3 ± 1.2	6.3 (3.8–9.8)	6.1 ± 0.9	6.0 (4.4–8.3)	0.510*
Anterior cingulate gyrus left	6.5 ± 1.3	6.3 (2.6–9.9)	6.8 ± 1.2	6.7 (4.0–10.1)	6.5 ± 1.0	6.4 (4.9–8.5)	0.251**
Middle cingulate gyrus total	11.5 ± 2.0	11.6 (7.1–16.7)	12.0 ± 2.2	11.7 (8.4–18.5)	11.8 ± 1.7	11.8 (9.1–15.6)	0.335*
Middle cingulate gyrus right	5.7 ± 1.0	5.6 (3.2–8.4)	6.0 ± 1.1	5.8 (4.5–9.7)	5.9 ± 0.9	5.9 (4.4–7.5)	0.464**
Middle cingulate gyrus left	5.8 ± 1.0	5.8 (3.8–8.3)	6.1 ± 1.1	5.8 (4.0–8.8)	5.9 ± 0.9	5.8 (4.6–8.1)	0.325*
Posterior cingulate gyrus total	9.0 ± 1.8	8.7 (6.0–14.4)	9.3 ± 1.8	8.9 (6.7–14.0)	9.0 ± 1.1	8.9 (7.1–12)	0.809**
Posterior cingulate gyrus right	4.5 ± 0.9	4.3 (2.9–7.1)	4.6 ± 0.9	4.4 (3.1–7.2)	4.4 ± 0.6	4.4 (3.4–6.3)	0.854**
Posterior cingulate gyrus left	4.6 ± 1.0	4.4 (2.7–7.3)	4.7 ± 1.0	4.5 (3.3–7.0)	4.6 ± 0.6	4.5 (3.4–6.4)	0.669**
Parahippocampal gyrus total	5.2 ± 1.0	5.2 (2.7–7.4)	5.3 ± 0.8	5.3 (4.1–7.1)	5.1 ± 0.6	5.0 (4.2–7.0)	0.507***
Parahippocampal gyrus right	2.5 ± 0.5	2.5 (1.3–3.5)	2.6 ± 0.4	2.6 (1.8–3.5)	2.5 ± 0.4	2.4 (1.8–3.5)	0.507*
Parahippocampal gyrus left	2.7 ± 0.5	2.7 (1.4–3.9)	2.7 ± 0.4	2.7 (1.9–3.7)	2.6 ± 0.3	2.6 (2.1–3.5)	0.606**
Insular total	28.4 ± 4.9	27.8 (17.3–41.2)	29.3 ± 4.3	28.9 (21.6–39.0)	27.9 ± 3.4	27.5 (20.5–37.7)	0.186*
Insular right	13.7 ± 2.3	13.3 (8.1–19.9)	14.2 ± 2.1	14.0 (10.5–19.9)	13.5 ± 1.6	13.3 (10.1–18.5)	0.133*
Insular left	14.8 ± 2.7	14.4 (8.8–21.3)	15.1 ± 2.3	14.5 (11.1–21.1)	14.4 ± 1.9	14.1 (10.4–19.5)	0.248*
Anterior insula total	8.2 ± 1.5	8.2 (4.2–11.8)	8.5 ± 1.4	8.4 (5.7–11.9)	8.3 ± 1.0	8.1 (5.9–11.2)	0.433*
Anterior insula right	4.1 ± 0.7	4.1 (2.2–5.8)	4.3 ± 0.7	4.3 (2.9–5.9)	4.1 ± 0.5	4.0 (2.9–5.4)	0.288*
Anterior insula left	4.1 ± 0.8	4.1 (1.9–6.1)	4.3 ± 0.7	4.2 (2.8–6.1)	4.2 ± 0.5	4.1 (3.0–5.8)	0.613***
Posterior insula total	4.3 ± 0.8	4.3 (2.6–6.9)	4.5 ± 0.7	4.4 (2.9–5.9)	4.3 ± 0.6	4.3 (2.9–5.6)	0.309*
Posterior insula right	2.2 ± 0.4	2.2 (1.4–3.3)	2.3 ± 0.4	2.2 (1.5–3.2)	2.2 ± 0.3	2.2 (1.6–2.9)	0.286**
Posterior insula left	2.1 ± 0.5	2.1 (1.2–3.5)	2.2 ± 0.4	2.1 (1.4–3.1)	2.1 ± 0.3	2.1 (1.3–2.8)	0.565***
Central operculum total	7.8 ± 1.5	7.5 (5.1–11.6)	8.1 ± 1.4	8.1 (5.8–11.9)	7.5 ± 1.2	7.3 (5.4–11.1)	0.079**
Central operculum right	3.7 ± 0.7	3.6 (2.4–5.8)	3.9 ± 0.7	3.7 (2.7–5.6)	3.6 ± 0.6	3.6 (2.6–5.4)	0.111**
Central operculum left	4.1 ± 0.8	3.9 (2.8–6.5)	4.2 ± 0.8	4.2 (3.0–6.5)	3.9 ± 0.7	3.7 (2.7–6.0)	0.140**
Frontal operculum total	3.9 ± 0.8	3.8 (2.2–6.1)	4.1 ± 0.7	4.0 (2.6–5.7)	3.9 ± 0.6	3.8 (2.3–5.2)	0.257**
Frontal operculum right	1.9 ± 0.4	1.8 (0.9–2.9)	2.0 ± 0.3	2.0 (1.3–2.8)	1.9 ± 0.3	1.8 (1.1–2.4)	0.102*
Frontal operculum left	2.1 ± 0.5	2.0 (1.1–3.2)	2.1 ± 0.4	2.0 (1.2–3.3)	2.0 ± 0.4	1.9 (1.2–3.0)	0.428**
Parietal operculum total	4.1 ± 1.0	4.0 (2.0–6.6)	4.2 ± 0.8	4.2 (1.7–6.0)	3.9 ± 0.8	3.8 (2.2–6.2)	0.257*
Parietal operculum right	1.8 ± 0.5	1.7 (1.0–2.8)	1.8 ± 0.5	1.8 (0.8–2.9)	1.7 ± 0.4	1.7 (0.9–3.1)	0.904*
Parietal operculum left	2.4 ± 0.6	2.3 (1.0–3.9)^a^	2.4 ± 0.5	2.4 (0.9–3.5)^a^	2.2 ± 0.5	2.1 (1.0–3.6)^b^	0.023**
Cerebellar Vermal Lobules I–V	3.0 ± 0.6	3.0 (1.8–4.4)	3.0 ± 0.6	3.0 (1.8–4.5)	3.0 ± 0.6	3.0 (1.7–4.7)	0.938*
Cerebellar Vermal Lobules VI–VII	1.6 ± 0.3	1.6 (0.9–2.5)	1.6 ± 0.3	1.6 (1.0–2.4)	1.7 ± 0.3	1.6 (0.8–2.4)	0.604*
Cerebellar Vermal Lobules VIII–X	2.6 ± 0.5	2.6 (1.3–3.9)	2.5 ± 0.5	2.5 (1.2–3.7)	2.6 ± 0.4	2.6 (1.6–3.7)	0.369*

*ANOVA; **Kruskal–Wallis test; ***Welch test.

^a–d^Within each row, groups sharing the same letter are not significantly different. Statistically significant values are shown in bold.

HC, healthy control; EM, episodic migraine; CM, chronic migraine.

However, given the large number of regional comparisons performed (*n* = 220), correction for multiple testing was applied using both Holm–Bonferroni (family-wise error rate control) and Benjamini–Hochberg (false discovery rate control) procedures. After these corrections, none of the regional volumetric differences remained statistically significant.

Overall, when rigorous multiple-comparison control was applied across all examined brain regions, no statistically significant volumetric differences were detected between EM, CM, and HC groups.

## Discussion

In this automated structural MRI study, we compared whole-brain volumetric measures among patients with episodic migraine (EM), chronic migraine (CM), and healthy controls (HC). The principal finding of the present analysis is that no statistically significant volumetric differences were detected between groups after rigorous correction for multiple comparisons across 220 brain regions. Although several frontal, temporal, and parietal regions demonstrated apparent differences in the initial uncorrected analyses, these did not remain significant following family-wise error rate (FWER) and false discovery rate (FDR) control. Accordingly, these regional trends should be interpreted as exploratory rather than confirmatory.

Our findings differ from several previous neuroimaging studies reporting regional gray matter alterations in migraine, including the study by Chen et al. (12). Multiple methodological factors may account for these discrepancies. First, the present study applied stringent correction for multiple comparisons across a large number of regions. While this approach substantially reduces the probability of Type I error, it simultaneously increases the risk of Type II error and may obscure subtle volumetric effects. Second, heterogeneity in imaging protocols—including magnetic field strength, spatial resolution, and segmentation pipelines—can materially influence volumetric estimates and inter-study comparability. Third, differences in clinical phenotyping, particularly regarding medication exposure, migraine chronicity definitions, and comorbid conditions, may further contribute to variability across studies. Consistent with recent meta-analyses, the structural neuroimaging literature in migraine remains heterogeneous and has not identified a single reproducible morphological signature (6, 7).

From a methodological perspective, our study employed a comprehensive whole-brain parcellation strategy rather than a hypothesis-driven region-of-interest (ROI) design. Whole-brain analyses provide unbiased anatomical coverage but necessitate stringent statistical correction when numerous comparisons are performed. Under such conservative thresholds, small regional effects related to migraine chronification may fail to reach statistical significance. Future investigations guided by strong *a priori* hypotheses may benefit from targeted ROI approaches, which could improve statistical sensitivity while maintaining appropriate control of false-positive findings.

An additional consideration is the potential dissociation between structural and functional alterations in migraine. Prior work in chronic pain populations suggests that persistent pain states may be more consistently associated with functional network reorganization than with robust structural abnormalities (16, 17). In this context, the absence of significant volumetric differences in the present cohort does not exclude the possibility of functional alterations within trigeminovascular and pain-processing networks in migraine. Rather, our findings support the possibility that structural effects—if present—are modest and methodologically sensitive.

To further aid interpretation, we report Cohen’s *d* effect sizes as exploratory measures (Supplementary Table S3). Across the analyzed regional set, effect sizes were predominantly small. For the CM vs. EM comparison, the median absolute effect size was approximately 0.21, with the large majority of regions showing |*d*| < 0.30 and no regions demonstrating large effects. For CM vs. HC, effect sizes were even smaller overall (median |*d*| ~ 0.08), indicating minimal volumetric separation between groups. These findings reinforce that, within the sensitivity limits of the present imaging protocol and analytical framework, potential structural differences associated with migraine chronification—if present—are likely modest in magnitude and methodologically sensitive.

Taken together, the present results suggest that migraine chronification may not be associated with large, robust volumetric brain changes detectable using conservative whole-brain correction strategies. This interpretation aligns with the growing recognition that previously reported morphometric differences in migraine may depend strongly on analytical choices, cohort characteristics, and imaging resolution (6–8).

Importantly, the absence of statistically significant volumetric differences after rigorous multiple-comparison correction should not be interpreted as a negative or inconclusive outcome. Well-powered null findings play a critical role in refining pathophysiological models by helping to constrain effect sizes and reduce publication bias in the neuroimaging literature. In the present study, the combination of adequate sample size, comprehensive whole-brain coverage, and stringent error control provides meaningful evidence that large or robust macrostructural differences between episodic and chronic migraine are unlikely under the current imaging conditions. When considered alongside the predominantly small effect sizes observed, our results suggest that previously reported morphometric alterations in migraine may be more modest, heterogeneous, or methodologically sensitive than often assumed. From a translational perspective, these findings support the possibility that functional network alterations, microstructural changes, or longitudinal dynamics—rather than cross-sectional volumetric differences—may represent more sensitive neurobiological markers of migraine chronification. Accordingly, the present null results contribute constructively to the field by narrowing plausible effect magnitudes and informing the design of future hypothesis-driven and longitudinal studies.

## Limitations

This study has several limitations that should be considered when interpreting the findings. First, imaging was performed using a 1.5-T MRI system. Although high-resolution isotropic T1-weighted images (1 × 1 × 1 mm) were used, the use of a 1.5-T field strength may provide lower contrast-to-noise ratio compared with contemporary 3-T systems and may reduce sensitivity for detecting very subtle morphometric differences. Therefore, small cortical effects related to migraine chronification may remain below the detection threshold of the present imaging protocol.

Second, the control group consisted of patients presenting with dizziness rather than asymptomatic community controls. Although none had a history of migraine, the potential influence of underlying vestibular or neurological conditions cannot be completely excluded.

Third, detailed data regarding medication exposure—including preventive therapies, acute treatments, and possible medication overuse—were not systematically available. Given the potential effects of medications on brain structure, this represents an important source of unmeasured variability.

Fourth, the study included only episodic migraine patients without aura, and the proportion of male participants was limited, which may affect generalizability. Fifth, the cross-sectional design precludes conclusions regarding causal relationships or the temporal evolution from episodic to chronic migraine.

From a methodological perspective, the use of a whole-brain exploratory approach with correction across 220 regions provides strong protection against Type I error but may increase the risk of Type II error and obscure small regional effects. Although the achieved sample size met the *a priori* power calculation, subtle volumetric differences below the detection threshold cannot be excluded. Finally, although an automated and standardized volBrain pipeline was used, formal manual visual inspection of segmentation outputs was not systematically performed, which represents a minor methodological limitation.

## Conclusion

In conclusion, after rigorous control for multiple comparisons, this study did not demonstrate significant brain volumetric differences between episodic migraine, chronic migraine, and control groups. These findings suggest that structural brain alterations reported in migraine may be smaller, more heterogeneous, or more methodologically sensitive than often assumed. Future studies incorporating higher-resolution imaging, longitudinal follow-up, detailed medication profiling, and hypothesis-driven regional analyses are warranted to further clarify the relationship between migraine chronification and brain structure.

## Data Availability

The original contributions presented in the study are included in the article/Supplementary material, further inquiries can be directed to the corresponding author.

## References

[1] Dai W Liu RH Qiu E Liu Y Chen Z Chen X . Cortical mechanisms in migraine. Mol Pain. (2021) 17:17448069211050246. doi: 10.1177/17448069211050246, 34806494 PMC8606910

[2] Bigal ME Lipton RB. Clinical course in migraine: conceptualizing migraine transformation. Neurology. (2008) 71:848–55. doi: 10.1212/01.wnl.0000325565.63526.d2, 18779513

[3] Scher AI Lipton RB Stewart W. Risk factors for chronic daily headache. Current Science Inc. (2002) 6:486–91. doi: 10.1007/s11916-002-0068-8, 12413408

[4] Katsarava Z Schneeweiss S Kurth T Kroener U Fritsche G Eikermann A . Incidence and predictors for chronicity of headache in patients with episodic migraine. Neurology. (2004) 62:788–90. doi: 10.1212/01.wnl.0000113747.18760.d2, 15007133

[5] Atraszkiewicz D. The processes underlying chronic migraine pathophysiology and its treatment with botulinum toxin type a. Neurol Clin Neurosci. (2021) 9:421–9. doi: 10.1111/ncn3.12551

[6] Chen ZH Cui YL Sun JT Li YT Zhang C Zhang YM . The brain structure and function abnormalities of migraineurs: a systematic review and neuroimaging meta-analysis. Front Neurol. (2022) 13:1022793. doi: 10.3389/fneur.2022.1022793, 36419535 PMC9676357

[7] Wang HZ Wang WH Shi HC Yuan CH. Is there a reliable brain morphological signature for migraine? J Headache Pain. (2020) 21:89. doi: 10.1186/s10194-020-01158-7, 32652927 PMC7353790

[8] Zhang X Zhou J Guo M Cheng S Chen Y Jiang N . A systematic review and meta-analysis of voxel-based morphometric studies of migraine. J Neurol. (2023) 270:152–70. doi: 10.1007/s00415-022-11363-w, 36098838

[9] Parisi P Belcastro V Verrotti A Striano P Kasteleijn-Nolst Trenitè DGA. “Ictal epileptic headache” and the revised international headache classification (ICHD-3) published in Cephalalgia 2018, vol. 38(1) 1-211: not just a matter of definition! Epilepsy Behav. (2018) 87:243–5. doi: 10.1016/j.yebeh.2018.07.018 30115602

[10] Ochs AL Ross DE Zannoni MD Abildskov TJ Bigler ED Alzheimer's Disease Neuroimaging Initiative. Comparison of automated brain volume measures obtained with NeuroQuant and FreeSurfer. J Neuroimaging. (2015) 25:721–7. doi: 10.1111/jon.12229, 25727700

[11] Manjón JV Coupé P. volBrain: an online MRI brain Volumetry system. Front Neuroinform. (2016) 10:30. doi: 10.3389/fninf.2016.00030, 27512372 PMC4961698

[12] Chen XY Chen ZY Dong Z Liu MQ Yu SY. Regional volume changes of the brain in migraine chronification. Neural Regen Res. (2020) 15:1701–8. doi: 10.4103/1673-5374.276360, 32209774 PMC7437590

[13] R Core Team. R: A Language and Environment for Statistical Computing. Vienna: R Foundation for Statistical Computing (2023).

[14] Holm S. A simple sequentially rejective multiple test procedure. Scand J Stat. (1979) 6:65–70.

[15] Benjamini Y Hochberg Y. Controlling the false discovery rate: a practical and powerful approach to multiple testing. J R Stat Soc Series B Stat Methodol. (1995) 57:289–300. doi: 10.1111/j.2517-6161.1995.tb02031.x

[16] Apkarian VA Hashmi JA Baliki MN. Pain and the brain: specificity and plasticity of the brain in clinical chronic pain. Pain. (2011) 152:S49–64. doi: 10.1016/j.pain.2010.11.010, 21146929 PMC3045648

[17] Loggia ML Edwards RR Kim J Vangel MG Wasan AD Gollub RL . Disentangling linear and nonlinear brain responses to evoked deep tissue pain. Pain. (2012) 153:2140–51. doi: 10.1016/j.pain.2012.07.014 22883925 PMC3445769

