# Supplementary material for: Comparison of brain volumes in episodic and chronic migraine using automated whole-brain volumetry
Source: Front Neurol. 2026 Mar 16;17:1772869. doi: 10.3389/fneur.2026.1772869 (PMC13033485; doi:10.3389/fneur.2026.1772869)
Supplement: Supplementary file 1 [file Table_1.DOCX]

Supplementary Material

Supplementary Table S1. Global brain volumetric measures (cm³) across healthy control, episodic migraine, and chronic migraine groups.

| VOLUMES (cm^3^) | HC | | EM | | CM | | p |
| --- | --- | --- | --- | --- | --- | --- | --- |
|  | mean ± SD | median (min-max) | mean ± SD | median (min-max) | mean ± SD | median (min-max) |  |
| White Matter (WM) | 533,8 ± 62,1 | 533,6 (384,2 - 703,7) | 531,7 ± 72,8 | 523,7 (389,4 – 749,0) | 532,1 ± 65,1 | 523,8 (385,4 - 690,6) | 0,985* |
| Grey Matter (GM) | 596,5 ± 88,3 | 584,5 (400,5 - 811,2) | 618,9 ± 90,0 | 605,1 (438,9 - 910,6) | 599,6 ± 71,0 | 591,9 (469,4 - 804,2) | 0,304* |
| Subcortical Grey Matter | 32,9 ± 7,3 | 30,6 (20,5 - 57,2) | 34,6 ± 7,0 | 33,7 (23,7 - 51,8) | 33,0 ± 5,1 | 33,0 (23,5 - 44,8) | 0,378*** |
| Cortical Grey Matter | 480,4 ± 75,2 | 477,6 (318,4 - 675,7) | 499,1 ± 79,3 | 486,5 (324,3 – 769,0) | 481,9 ± 59,7 | 476,3 (378,7 - 648,3) | 0,309* |
| Cerebellar Grey Matter | 83,2 ± 10,5 | 81,7 (56 - 107,8) | 85,2 ± 10,7 | 84,5 (59,5 - 112,4) | 84,6 ± 10,5 | 84,7 (60,0 - 111,1) | 0,593* |
| Cerebro Spinal Fluid (CSF) | 151,7 ± 49,0 | 142,7 (80,3 - 358,2) | 140,5 ± 41,0 | 133,5 (90,2 - 358,7) | 145,7 ± 32,3 | 142,6 (82,2 - 222,2) | 0,235*** |
| Brain (WM+GM) | 1130,3 ± 113,2 | 1142 (883,5 - 1408,3) | 1150,7 ± 128,9 | 1155,8 (828,4 - 1659,6) | 1131,7 ± 100,0 | 1142,3 (898,1 – 1367,0) | 0,572* |
| Intracranial Cavity (IC) | 1299,9 ± 117,1 | 1300,1 (1077,3 - 1647,2) | 1309,5 ± 130,1 | 1311 (1120,5 - 1864,1) | 1295,4 ± 114,3 | 1295,8 (1040,7 - 1565,5) | 0,946*** |
| Cerebrum total | 1003,3 ± 105,1 | 1009,2 (791,6 - 1270,9) | 1022 ± 119,0 | 1023,5 (709,7 - 1493,3) | 1003,2 ± 91,2 | 1006,6 (793,5 - 1210,2) | 0,553* |
| Cerebrum right | 503,1 ± 52,9 | 506,1 (396,5 - 635,7) | 511,8 ± 60,3 | 511,5 (349,2 - 750,8) | 502,0 ± 45,4 | 502,0 (398,7 – 607,0) | 0,717*** |
| Cerebrum left | 500,3 ± 52,3 | 503 (395,1 - 635,2) | 510,2 ± 58,8 | 511,2 (360,5 - 742,4) | 501,2 ± 45,9 | 501,0 (394,8 - 603,2) | 0,540* |
| Cerebrum WM total | 490 ± 57,4 | 487 (353,4 - 654,5) | 488,3 ± 66,3 | 481,4 (342,1 - 681,1) | 488,2 ± 59,8 | 476,6 (356,2 - 635,9) | 0,983* |
| Cerebrum WM right | 247,7 ± 28,8 | 245,3 (179,3 - 330,5) | 246,7 ± 35,5 | 242,2 (170,8 - 355,2) | 246,3 ± 30,4 | 241,1 (180,5 - 320,8) | 0,969* |
| Cerebrum WM left | 242,3 ± 28,7 | 240 (174,2 – 324,0) | 241,5 ± 31,6 | 240,2 (171,3 - 336,9) | 241,9 ± 29,6 | 236,1 (175,7 - 315,1) | 0,991* |
| Cerebrum GM total | 513,3 ± 81,1 | 506,8 (342,9 – 724,0) | 533,8 ± 84,0 | 515,9 (367,6 - 812,2) | 515,0 ± 63,3 | 509,5 (402,1 - 693,1) | 0,292* |
| Cerebrum GM right | 255,3 ± 40,8 | 251,1 (172,1 - 360,5) | 265,1 ± 42,3 | 255,9 (178,4 - 406,6) | 255,6 ± 31,2 | 254,6 (201,6 - 345,1) | 0,312* |
| Cerebrum GM left | 257,9 ± 40,4 | 253,6 (170,8 - 363,6) | 268,7 ± 42,2 | 260,0 (189,2 - 405,5) | 259,3 ± 32,3 | 255,5 (199,6 - 347,9) | 0,275* |
| Cerebellum total | 119,8 ± 11,4 | 121,1 (81,6 - 142,5) | 121,6 ± 12,8 | 123,0 (91,0 - 159,1) | 121,2 ± 11,6 | 121,9 (87,1 - 148,1) | 0,691* |
| Cerebellum right | 60,1 ± 5,8 | 60,6 (41,4 - 71,3) | 61,2 ± 6,5 | 61,9 (45,1 - 80,9) | 60,9 ± 6,0 | 61,0 (43,3 - 74,3) | 0,696*** |
| Cerebellum left | 59,6 ± 5,8 | 60 (40,2 - 71,2) | 60,3 ± 6,4 | 60,7 (45,8 - 78,2) | 60,2 ± 5,6 | 60,6 (43,8 - 73,8) | 0,792* |
| Cerebellum WM total | 43,7 ± 6,9 | 44,7 (29,7 - 61,6) | 43,5 ± 8,2 | 42,7 (29,0 - 67,9) | 43,8 ± 7,4 | 43,9 (29,2 - 62,1) | 0,966* |
| Cerebellum WM right | 22,9 ± 3,3 | 23,5 (15,9 - 30,5) | 23 ± 4,3 | 23,2 (15,0 – 38,0) | 23,1 ± 4,0 | 23,2 (15,5 - 33,3) | 0,954*** |
| Cerebellum WM left | 20,8 ± 3,7 | 20,8 (13,3 - 31,1) | 20,5 ± 4,3 | 19,6 (14,0 - 30,7) | 20,7 ± 3,5 | 20,7 (13,1 – 30,0) | 0,922* |
| Cerebellum GM total | 83,2 ± 10,5 | 81,7 (56 - 107,8) | 85,2 ± 10,7 | 84,5 (59,5 - 112,4) | 84,6 ± 10,5 | 84,7 (60,0 - 111,1) | 0,593* |
| Cerebellum GM right | 37,2 ± 4,8 | 36,7 (25 - 49,3) | 38,3 ± 4,9 | 38,2 (28,5 - 49,6) | 37,8 ± 5,0 | 38,0 (25,3 - 50,1) | 0,483* |
| Cerebellum GM left | 38,8 ± 5,1 | 38,2 (24,9 - 49,4) | 39,8 ± 5,3 | 40,3 (26,3 - 53,1) | 39,5 ± 4,9 | 39,2 (29,1 - 51,1) | 0,587* |
| Vermis | 7,2 ± 1,1 | 7,0 (4,5 - 9,9) | 7,1 ± 1,0 | 7,0 (4,7 - 9,7) | 7,3 ± 1,1 | 7,4 (5,2 - 9,9) | 0,651* |
| Brainstem | 17,9 ± 1,6 | 17,8 (14,7 - 21,3) | 18,3 ± 2,3 | 18,3 (14,3 – 24,0) | 18,0 ± 1,7 | 17,9 (14,0 - 22,7) | 0,626** |

*ANOVA, **Welch test, ***Kruskal Wallis Test

HC: Healthy Control, EM: Episodic migraine, CM: Chronic migraine

Supplementary Table S2. Basal ganglia volumetric comparisons between groups.

| VOLUMES (cm^3^) | HC | | EM | | CM | | p |
| --- | --- | --- | --- | --- | --- | --- | --- |
|  | mean ± SD | median (min-max) | mean ± SD | median (min-max) | mean ± SD | median (min-max) |  |
| Accumbens total | 0,3 ± 0,2 | 0,3 (0,0 - 0,9) | 0,3 ± 0,2 | 0,3 (0,1 - 0,8) | 0,3 ± 0,2 | 0,3 (0,0 - 0,9) | 0,284** |
| Amygdala total | 1,9 ± 0,6 | 1,9 (0,6 - 3,3) | 2,0 ± 0,5 | 2,0 (0,8 - 3,5) | 1,9 ± 0,5 | 1,8 (0,7 - 2,8) | 0,408* |
| Amygdala right | 0,9 ± 0,3 | 0,9 (0,3 - 1,8) | 1,0 ± 0,3 | 1,0 (0,4 - 1,7) | 1,0 ± 0,2 | 1,0 (0,4 - 1,5) | 0,539* |
| Amygdala left | 0,9 ± 0,3 | 0,9 (0,2 - 1,6) | 1,0 ± 0,3 | 1,0 (0,4 – 2,0) | 0,9 ± 0,3 | 0,9 (0,3 - 1,6) | 0,122* |
| Basal Forebrain total | 0,7 ± 0,1 | 0,7 (0,3 - 0,9) | 0,7 ± 0,1 | 0,7 (0,4 – 1,0) | 0,7 ± 0,1 | 0,7 (0,4 – 1,0) | 0,137* |
| Basal Forebrain right | 0,3 ± 0,1 | 0,3 (0,2 - 0,4) | 0,4 ± 0,1 | 0,4 (0,2 - 0,5) | 0,3 ± 0,1 | 0,3 (0,2 - 0,5) | 0,117* |
| Basal Forebrain left | 0,3 ± 0,1 | 0,3 (0,2 - 0,5) | 0,4 ± 0,1 | 0,4 (0,2 - 0,5) | 0,3 ± 0,1 | 0,3 (0,2 - 0,6) | 0,170** |
| Caudate total | 5,7 ± 1,6 | 5,3 (3,2 - 11,2) | 5,9 ± 1,6 | 5,5 (3,4 - 10,6) | 5,7 ± 1,2 | 5,5 (3,4 - 8,9) | 0,782** |
| Caudate right | 2,8 ± 0,8 | 2,5 (1,5 - 5,5) | 2,9 ± 0,9 | 2,6 (1,7 - 5,4) | 2,7 ± 0,6 | 2,7 (1,6 - 4,2) | 0,883** |
| Caudate left | 2,9 ± 0,8 | 2,7 (1,6 - 5,7) | 3,1 ± 0,8 | 2,9 (1,7 - 5,2) | 2,9 ± 0,6 | 2,9 (1,7 - 4,7) | 0,730** |
| Hippocampus total | 7,9 ± 1,4 | 7,9 (4,9 - 11,8) | 8,2 ± 1,3 | 8,2 (5,1 - 11,1) | 7,7 ± 1,2 | 7,5 (5,0 - 10,9) | 0,188* |
| Hippocampus right | 3,9 ± 0,8 | 3,8 (2,6 - 5,9) | 4,0 ± 0,7 | 4,0 (2,4 - 5,7) | 3,8 ± 0,6 | 3,8 (2,2 - 5,6) | 0,390* |
| Hippocampus left | 3,9 ± 0,7 | 4,0 (2,3 - 5,9) | 4,1 ± 0,6 | 4,1 (2,7 - 5,6) | 3,9 ± 0,6 | 3,8 (2,8 - 5,4) | 0,094* |
| Pallidum total | 2,7 ± 0,8 | 2,7 (1,2 - 5,3) | 2,7 ± 0,7 | 2,6 (1,2 - 4,9) | 2,9 ± 0,7 | 2,9 (1,7 - 4,7) | 0,266* |
| Pallidum right | 1,5 ± 0,4 | 1,5 (0,5 - 2,7) | 1,4 ± 0,4 | 1,4 (0,7 - 2,5) | 1,5 ± 0,4 | 1,6 (0,6 - 2,6) | 0,509* |
| Pallidum left | 1,2 ± 0,4 | 1,2 (0,6 - 2,5) | 1,2 ± 0,4 | 1,2 (0,6 - 2,4) | 1,3 ± 0,3 | 1,3 (0,8 - 2,1) | 0,074** |
| Putamen total | 3,0 ± 1,7 | 2,7 (0,5 - 8,1) | 3,5 ± 1,9 | 3,4 (0,7 - 8,6) | 2,9 ± 1,6 | 2,5 (0,6 - 7,4) | 0,157** |
| Putamen right | 1,4 ± 0,8 | 1,2 (0,2 - 3,7) | 1,7 ± 0,9 | 1,6 (0,4 - 4,1) | 1,3 ± 0,8 | 1,2 (0,3 - 3,6) | 0,101** |
| Putamen left | 1,6 ± 1,0 | 1,5 (0,2 - 4,4) | 1,8 ± 1,0 | 1,9 (0,2 - 4,6) | 1,5 ± 0,8 | 1,3 (0,3 - 3,8) | 0,186* |
| Thalamus total | 10,8 ± 3,1 | 10,1 (5,4 - 19,6) | 11,4 ± 3,2 | 10,3 (6,6 - 19,8) | 11,1 ± 2,4 | 11,3 (6,7 - 18,9) | 0,427** |
| Thalamus right | 5,3 ± 1,6 | 4,9 (2,6 - 10,1) | 5,6 ± 1,6 | 5,3 (3,3 - 10,4) | 5,5 ± 1,3 | 5,4 (3,2 - 10,2) | 0,328** |
| Thalamus left | 5,5 ± 1,5 | 5,3 (2,8 - 9,4) | 5,7 ± 1,6 | 5,3 (3,3 - 9,4) | 5,7 ± 1,2 | 5,7 (3,0 - 8,7) | 0,596** |

*ANOVA, **Kruskal Wallis Test

HC: Healthy Control, EM: Episodic migraine, CM: Chronic migraine

Supplementary Table S3. Regional volumetric effect sizes (Cohen’s d).

| Brain region | EM vs HC (d) | CM vs HC (d) | CM vs EM (d) |
| --- | --- | --- | --- |
| Ventral DC total | 0.100 | -0.105 | -0.211 |
| Ventral DC right | 0.000 | -0.200 | -0.200 |
| Ventral DC left | 0.200 | 0.000 | -0.200 |
| Frontal total | 0.309 | 0.099 | -0.239 |
| Frontal right | 0.297 | 0.094 | -0.232 |
| Frontal left | 0.317 | 0.103 | -0.242 |
| Frontal pole total | 0.552 | 0.296 | -0.286 |
| Frontal pole right | 0.375 | 0.133 | -0.267 |
| Frontal pole left | 0.571 | 0.286 | -0.286 |
| Gyrus rectus total | 0.374 | 0.133 | -0.235 |
| Gyrus rectus right | 0.222 | 0.000 | -0.222 |
| Gyrus rectus left | 0.444 | 0.250 | -0.222 |
| Opercular inf. frontal gyrus total | 0.333 | 0.181 | -0.182 |
| Opercular inf. frontal gyrus right | 0.153 | 0.000 | -0.167 |
| Opercular inf. frontal gyrus left | 0.462 | 0.362 | -0.165 |
| Orbital inf. frontal gyrus total | 0.326 | 0.000 | -0.400 |
| Orbital inf. frontal gyrus right | 0.281 | 0.000 | -0.282 |
| Orbital inf. frontal gyrus left | 0.250 | 0.000 | -0.250 |
| Triangular inf. frontal gyrus total | 0.319 | 0.091 | -0.239 |
| Triangular inf. frontal gyrus right | 0.267 | 0.153 | -0.142 |
| Triangular inf. frontal gyrus left | 0.308 | 0.000 | -0.307 |
| Medial frontal cortex total | 0.308 | 0.000 | -0.330 |
| Medial frontal cortex right | 0.333 | 0.000 | -0.333 |
| Medial frontal cortex left | 0.285 | 0.000 | -0.284 |
| Middle frontal gyrus total | 0.256 | 0.072 | -0.199 |
| Middle frontal gyrus right | 0.277 | 0.070 | -0.225 |
| Middle frontal gyrus left | 0.226 | 0.107 | -0.133 |
| Anterior orbital gyrus total | 0.235 | 0.266 | 0.000 |
| Anterior orbital gyrus right | 0.400 | 0.220 | -0.222 |
| Anterior orbital gyrus left | 0.222 | 0.250 | 0.000 |
| Lateral orbital gyrus total | 0.375 | 0.125 | -0.250 |
| Lateral orbital gyrus right | 0.250 | 0.000 | -0.250 |
| Lateral orbital gyrus left | 0.500 | 0.250 | -0.250 |
| Medial orbital gyrus total | 0.193 | 0.066 | -0.138 |
| Medial orbital gyrus right | 0.117 | 0.000 | -0.125 |
| Medial orbital gyrus left | 0.265 | 0.000 | -0.286 |
| Posterior orbital gyrus total | 0.174 | 0.000 | -0.205 |
| Posterior orbital gyrus right | 0.167 | 0.195 | 0.000 |
| Posterior orbital gyrus left | 0.182 | 0.000 | -0.197 |
| Precentral gyrus total | 0.082 | 0.030 | -0.057 |
| Precentral gyrus right | 0.056 | 0.059 | 0.000 |
| Precentral gyrus left | 0.103 | 0.000 | -0.111 |
| Precentral gyrus medial segment total | 0.222 | 0.000 | -0.235 |
| Precentral gyrus medial segment right | 0.000 | -0.200 | -0.200 |
| Precentral gyrus medial segment left | 0.200 | 0.000 | -0.222 |
| Subcallosal area total | 0.153 | 0.000 | -0.167 |
| Subcallosal area right | 0.000 | 0.000 | 0.000 |
| Subcallosal area left | 0.000 | -0.333 | -0.333 |
| Sup. frontal gyrus total | 0.329 | 0.110 | -0.227 |
| Sup. frontal gyrus right | 0.266 | 0.091 | -0.186 |
| Sup. frontal gyrus left | 0.319 | 0.120 | -0.208 |
| Sup. frontal gyrus medial segment total | 0.359 | 0.114 | -0.262 |
| Sup. frontal gyrus medial segment right | 0.273 | 0.105 | -0.189 |
| Sup. frontal gyrus medial segment left | 0.381 | 0.105 | -0.299 |
| Supplementary motor cortex total | 0.200 | 0.071 | -0.143 |
| Supplementary motor cortex right | 0.118 | 0.000 | -0.124 |
| Supplementary motor cortex left | 0.125 | 0.000 | -0.133 |
| Temporal total | 0.212 | -0.094 | -0.330 |
| Temporal right | 0.167 | -0.128 | -0.318 |
| Temporal left | 0.252 | -0.055 | -0.329 |
| Fusiform gyrus total | 0.214 | 0.000 | -0.239 |
| Fusiform gyrus right | 0.138 | -0.079 | -0.229 |
| Fusiform gyrus left | 0.286 | 0.077 | -0.231 |
| Planum polare total | 0.361 | 0.195 | -0.222 |
| Planum polare right | 0.333 | 0.000 | -0.394 |
| Planum polare left | 0.333 | 0.000 | -0.394 |
| Planum temporale total | 0.143 | -0.286 | -0.429 |
| Planum temporale right | 0.000 | 0.000 | 0.000 |
| Planum temporale left | 0.250 | -0.250 | -0.500 |
| Inf. temporal gyrus total | 0.156 | -0.149 | -0.325 |
| Inf. temporal gyrus right | 0.100 | -0.169 | -0.284 |
| Inf. temporal gyrus left | 0.250 | -0.056 | -0.341 |
| Middle temporal gyrus total | 0.139 | -0.066 | -0.214 |
| Middle temporal gyrus right | 0.140 | -0.124 | -0.272 |
| Middle temporal gyrus left | 0.204 | 0.000 | -0.208 |
| Sup. temporal gyrus total | 0.217 | 0.000 | -0.243 |
| Sup. temporal gyrus right | 0.148 | -0.086 | -0.248 |
| Sup. temporal gyrus left | 0.191 | 0.100 | -0.095 |
| Transverse temporal gyrus total | -0.180 | -0.167 | 0.000 |
| Transverse temporal gyrus right | -0.333 | 0.000 | 0.333 |
| Transverse temporal gyrus left | -0.281 | -0.564 | -0.333 |
| Temporal pole total | 0.278 | -0.096 | -0.391 |
| Temporal pole right | 0.316 | -0.060 | -0.397 |
| Temporal pole left | 0.222 | -0.125 | -0.352 |
| Parietal total | 0.241 | 0.090 | -0.169 |
| Parietal right | 0.218 | 0.074 | -0.158 |
| Parietal left | 0.260 | 0.117 | -0.161 |
| Angular gyrus total | 0.242 | 0.061 | -0.184 |
| Angular gyrus right | 0.095 | -0.056 | -0.142 |
| Angular gyrus left | 0.388 | 0.118 | -0.263 |
| Postcentral gyrus total | 0.178 | -0.039 | -0.210 |
| Postcentral gyrus right | 0.133 | 0.000 | -0.138 |
| Postcentral gyrus left | 0.199 | 0.000 | -0.193 |
| Postcentral gyrus medial segment total | 0.000 | 0.000 | 0.000 |
| Postcentral gyrus medial segment right | -0.500 | 0.000 | 0.500 |
| Postcentral gyrus medial segment left | 0.396 | 0.500 | 0.000 |
| Precuneus total | 0.256 | 0.000 | -0.292 |
| Precuneus right | 0.300 | 0.056 | -0.284 |
| Precuneus left | 0.200 | 0.000 | -0.227 |
| Sup. parietal lobule total | 0.178 | 0.211 | 0.000 |
| Sup. parietal lobule right | 0.226 | 0.138 | -0.110 |
| Sup. parietal lobule left | 0.176 | 0.258 | 0.057 |
| Supramarginal gyrus total | 0.250 | 0.117 | -0.151 |
| Supramarginal gyrus right | 0.286 | 0.154 | -0.143 |
| Supramarginal gyrus left | 0.194 | 0.073 | -0.142 |
| Occipital total | 0.107 | -0.011 | -0.123 |
| Occipital right | 0.094 | -0.064 | -0.159 |
| Occipital left | 0.114 | 0.040 | -0.077 |
| Calcarine cortex total | 0.071 | 0.000 | -0.071 |
| Calcarine cortex left | 0.000 | 0.000 | 0.000 |
| Cuneus total | 0.190 | 0.000 | -0.200 |
| Cuneus right | 0.000 | -0.153 | -0.167 |
| Cuneus left | 0.000 | 0.000 | 0.000 |
| Lingual gyrus total | 0.091 | 0.000 | -0.091 |
| Lingual gyrus right | 0.090 | -0.087 | -0.190 |
| Lingual gyrus left | 0.080 | 0.080 | 0.000 |
| Occipital fusiform gyrus total | 0.105 | 0.111 | 0.000 |
| Occipital fusiform gyrus right | 0.364 | 0.200 | -0.182 |
| Occipital fusiform gyrus left | 0.167 | 0.181 | 0.000 |
| Inf. occipital gyrus total | 0.000 | -0.104 | -0.102 |
| Inf. occipital gyrus right | 0.083 | -0.099 | -0.180 |
| Inf. occipital gyrus left | -0.091 | -0.190 | -0.095 |
| Middle occipital gyrus total | 0.231 | 0.111 | -0.138 |
| Middle occipital gyrus right | 0.172 | 0.000 | -0.171 |
| Middle occipital gyrus left | 0.260 | 0.190 | -0.083 |
| Sup. occipital gyrus total | 0.083 | 0.000 | -0.090 |
| Sup. occipital gyrus right | 0.000 | -0.328 | -0.277 |
| Sup. occipital gyrus left | 0.182 | 0.000 | -0.167 |
| Occipital pole total | -0.100 | -0.095 | 0.000 |
| Occipital pole right | -0.200 | -0.200 | 0.000 |
| Occipital pole left | 0.000 | 0.000 | 0.000 |
| Limbic total | 0.205 | 0.031 | -0.211 |
| Limbic right | 0.194 | 0.031 | -0.195 |
| Limbic left | 0.236 | 0.030 | -0.248 |
| Entorhinal area total | 0.141 | 0.000 | -0.167 |
| Entorhinal area right | 0.000 | 0.000 | 0.000 |
| Entorhinal area left | 0.250 | 0.000 | -0.284 |
| Anterior cingulate gyrus total | 0.212 | 0.000 | -0.255 |
| Anterior cingulate gyrus right | 0.239 | 0.089 | -0.189 |
| Anterior cingulate gyrus left | 0.239 | 0.000 | -0.272 |
| Middle cingulate gyrus total | 0.238 | 0.161 | -0.102 |
| Middle cingulate gyrus right | 0.286 | 0.210 | -0.100 |
| Middle cingulate gyrus left | 0.286 | 0.105 | -0.200 |
| Posterior cingulate gyrus total | 0.167 | 0.000 | -0.202 |
| Posterior cingulate gyrus right | 0.111 | -0.130 | -0.263 |
| Posterior cingulate gyrus left | 0.100 | 0.000 | -0.122 |
| Parahippocampal gyrus total | 0.110 | -0.121 | -0.284 |
| Parahippocampal gyrus right | 0.220 | 0.000 | -0.250 |
| Parahippocampal gyrus left | 0.000 | -0.242 | -0.284 |
| Insular total | 0.195 | -0.118 | -0.362 |
| Insular right | 0.227 | -0.101 | -0.376 |
| Insular left | 0.119 | -0.171 | -0.333 |
| Anterior insula total | 0.206 | 0.078 | -0.165 |
| Anterior insula right | 0.286 | 0.000 | -0.330 |
| Anterior insula left | 0.265 | 0.149 | -0.165 |
| Posterior insula total | 0.265 | 0.000 | -0.307 |
| Posterior insula right | 0.250 | 0.000 | -0.284 |
| Posterior insula left | 0.220 | 0.000 | -0.284 |
| Central operculum total | 0.206 | -0.220 | -0.461 |
| Central operculum right | 0.286 | -0.153 | -0.461 |
| Central operculum left | 0.125 | -0.266 | -0.400 |
| Frontal operculum total | 0.265 | 0.000 | -0.307 |
| Frontal operculum right | 0.281 | 0.000 | -0.333 |
| Frontal operculum left | 0.000 | -0.220 | -0.250 |
| Parietal operculum total | 0.110 | -0.220 | -0.375 |
| Parietal operculum right | 0.000 | -0.220 | -0.222 |
| Parietal operculum left | 0.000 | -0.362 | -0.400 |
| Cerebellar Vermal Lobules I-V | 0.000 | 0.000 | 0.000 |
| Cerebellar Vermal Lobules VI-VII | 0.000 | 0.333 | 0.333 |
| Cerebellar Vermal Lobules VIII-X | -0.200 | 0.000 | 0.222 |
